# Direct-write of tungsten-carbide nanoSQUIDs based on focused ion beam induced deposition

**DOI:** 10.1039/d2na00602b

**Published:** 2022-09-26

**Authors:** Fabian Sigloch, Soraya Sangiao, Pablo Orús, José M. de Teresa

**Affiliations:** Instituto de Nanociencia y Materiales de Aragon (INMA), Universidad de Zaragoza-CSIC 50009 Zaragoza Spain deteresa@unizar.es; Laboratorio de Microscopías Avanzadas (LMA), Universidad de Zaragoza 50018 Spain

## Abstract

NanoSQUIDs are quantum sensors that excel in detecting a small change in magnetic flux with high sensitivity and high spatial resolution. Here, we employ resist-free direct-write Ga^+^ Focused Ion Beam Induced Deposition (FIBID) techniques to grow W–C nanoSQUIDs, and we investigate their electrical response to changes in the magnetic flux. Remarkably, FIBID allows the fast (3 min) growth of 700 nm × 300 nm nanoSQUIDs based on narrow nanobridges (50 nm wide) that act as Josephson junctions. Albeit the SQUIDs exhibit a comparatively low modulation depth and obtain a high inductance, the observed transfer coefficient (output voltage to magnetic flux change) is comparable to other SQUIDs (up to 1300 μV/*Φ*_0_), which correlates with the high resistivity of W–C in the normal state. We discuss here the potential of this approach to reduce the active area of the nanoSQUIDs to gain spatial resolution as well as their integration on cantilevers for scanning-SQUID applications.

## Abstract 2

NanoSQUIDs are quantum sensors that excel in detecting a change in magnetic flux with high sensitivity and high spatial resolution. Here, we employ Ga^+^ focused ion beam induced deposition techniques to grow W–C nanoSQUIDs and we investigate their electrical response.

## Graphical abstract

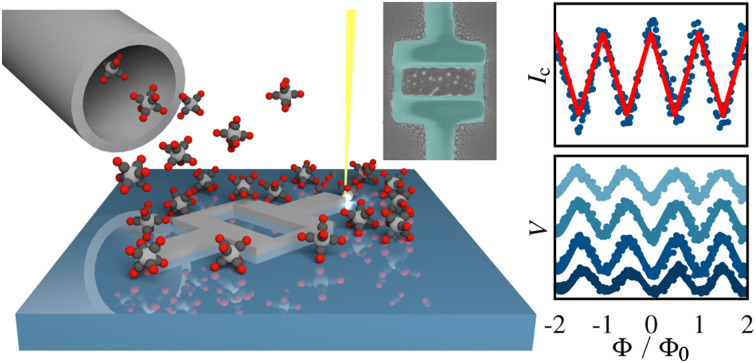

## 1 Introduction

**Fig. 1:**
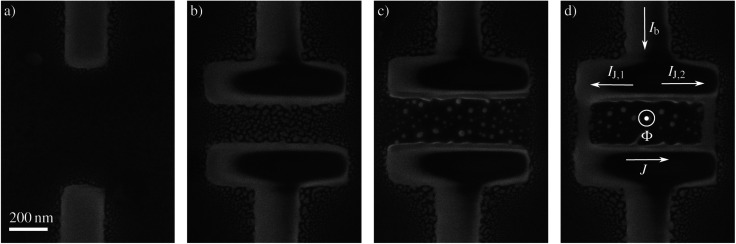
SEM images of the fabrication procedure of a W–C-SQUID by FIBID. (a) First the leads connecting the SQUID to the Au leads are deposited. (b) Secondly, two rectangular shapes are deposited. (c) In the third step, the halo residing in the inner loop area is removed by a short step of FIB milling. (d) In the last step, narrow (50 nm wide) nanobridges are grown.

Direct current- (dc-) Superconducting Quantum Interference Devices (SQUIDs) are magnetic flux sensors that attain an unrivaled sensitivity^1,2^ by exploiting the physical effects of magnetic flux quantization^3^ and Josephson effect.^4^ A dc-SQUID consists of a superconducting ring intersected by two Josephson Junctions (JJs), one on either side (Fig. 1d). A JJ is formed by a superconductor (S) interrupted by either an insulator (I), a normal metal (N) or a weaker superconductor (s) resulting, respectively, in a SIS-, SNS- or SsS-junction that is capable of carrying a superconducting Josephson current, *I*_J_.

The sensitivity of SQUIDs is limited by the flux noise, *S*_*Φ*_, and the SQUID inductance, *L*. The flux noise can be reduced by downsizing of the dimensions of the JJs,^5^ whereas the inductance can be reduced by decreasing the size of the effective inner SQUID area.^6^ A lot of effort has been brought forth and various methods have been developed to fabricate nanoSQUIDs with ever lower geometrical dimensions and thus higher sensitivity.^7–9^

While SQUIDs with SIS- or SNS-junctions commonly require a sandwich-type structure composed of multiple layers, SQUIDs based on SsS-junctions are planar and fabricated in a single layer. In this approach the JJs are realized by two nanobridges in the SQUID loop, forming regions with lower *I*_c_ in the superconducting material. The low thickness of the planar nanoSQUIDs makes them insusceptible to in-plane fields and enables for good coupling to magnetic nanoparticles. However, the dissipation of heat in the normal-conducting state of the nanobridges yields a hysteretic *I*–*V* characteristic in materials with high *I*_c_. Generally, the kinetic inductance, *L*_kin_ is high and can dominate the total inductance of the SQUID.^2^

Conventionally, the design for a nanoSQUID is transferred to a resist by means of Electron Beam Lithography (EBL). The structure can either be deposited *via* evaporation of a superconducting material followed by lift-off or etched from a previously patterned superconducting film.^10^ Processes based on EBL are well established and allow for complicated geometries with linewidths down to 30 nm.^5^ NanoSQUIDs based on nanobridges have been fabricated with an inner loop area of down to 200 nm × 100 nm and a flux sensitivity of 
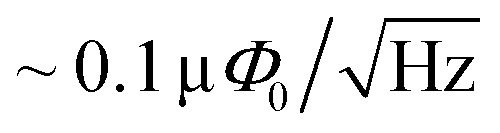
 by means of EBL.^10^ However, the resist-based EBL process requires multiple fabrication steps making it a time-consuming approach. A homogeneous film of the resist is obtained by spin-coating, requiring a large, flat substrate. Furthermore, the resulting structures are not perfectly symmetric and suffer from irregular edges.^11^

Novel, sophisticated fabrication methods such as variable thickness Dayem Bridges (DBs),^12,13^ superconducting Nb/Al bilayers^14^ and normal-conducting heat-sinks^9^ could further increase the sensitivity, but also add to the complexity of the fabrication process. The currently smallest, most sensitive nanoSQUIDs are based on a complicated process of directional evaporation of a superconducting material onto a pulled quartz tube. Vasyukov *et al.* fabricated a circular SQUID with a diameter of 50 nm, resulting in an inductance of 5.8 pH and a flux sensitivity below 
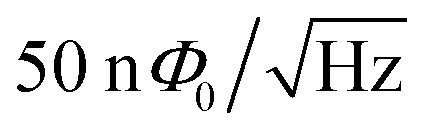
 making it capable of the detection of the spin of a single electron.^15^

A different approach to creating superconducting devices using direct-write techniques is to start from a superconducting thin film and perform a FIB irradiation process to locally modify the electronic properties. This approach has allowed, for example, for the creation of high-quality Josephson superconducting tunnel junctions by irradiation with a focused He^+^ ion beam.^16^ NanoSQUIDs with a DB-width of 30 nm and a loop size of 1 μm were fabricated by Ga^+^ FIB milling of a previously patterned Nb film in 1980 for the first time.^5^ Recently, M. Wyss *et al.* used this technique to fabricate a SQUID on the tip of a capped AFM cantilever with a flux sensitivity of 
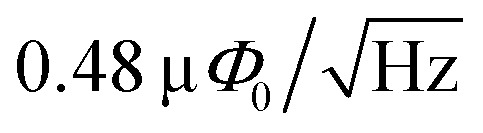
.^17^ However, the Nb in the DBs deteriorates due to the implantation of Ga and amorphization that occurs in the surface and up to 30 nm below it.

An alternative to resist-based techniques or directional evaporation are direct-write techniques, such as Focused Electron/Ion Induced Deposition (FEBID/FIBID), which constitute versatile techniques for the fabrication of nanostructures on substrates of arbitrary size and topography.^18,19^ These techniques do not require the use of a resist and the entire nanostructure can be fabricated in a single step. Typically performed in either dedicated FIB instruments or in FIB/Scanning Electron Microscope (SEM) equipments, which host columns of both ions and electrons, the procedure begins by introducing a gaseous precursor containing the element of interest into the process chamber, which then adsorbs on the substrate. Upon local irradiation of the adsorbed molecules with the focused beam, the precursor is decomposed into a non-volatile constituent, which permanently remains deposited on the surface, and into volatile by-products that are pumped away by the vacuum system of the instrument. The resulting deposit is patterned following the shape of the scan traced by the beam. In the case of FIBID, concurrently with the deposition of the desired material, the FIB modifies the exposed material by ion implantation, amorphization and sputtering. In absence of a precursor gas these effects can be used to locally modify the physical properties of a given sample or to locally remove material by milling.^20,21^

The ability of direct-write techniques to pattern very small features, and existing reports of JJ fabrication by FEBID^22^ and FIBID,^23^ make these techniques very promising for the fabrication of nanoSQUIDs in a single writing step.

The superconducting properties of W–C fabricated by Ga^+^ FIB irradiation of the commercially available precursor gas W(CO)_6_ are well studied.^24^ Planar Ga^+^ FIBID W–C deposits exhibit a critical temperature of *T*_c_ = 4–5 K,^25–28^ an upper critical magnetic field of *B*_c2_ = 7–8.5 T^29–31^ and a critical current density of *J*_c_ = 0.01–0.1 MA cm^−2^.^26–28^ The London penetration depth is reported to be *λ*_L_ = 850 nm^32,33^ and the superconducting coherence length *ξ* = 6–9 nm.^27,32–34^ Nanostructures with linewidths down to 50 nm can be patterned with high precision and reproducibility.^27^

Several remarkable applications for superconducting nanodevices fabricated by FIBID/FEBID have been reported thus far and are worth mentioning. W–C deposits have been used to induce proximity superconducting effects on other materials^35^ and to study the spin polarization of magnetic materials in Andreev contacts.^36^ Besides, narrow W–C nanowires fabricated by Ga^+^ FIBID have been found to sustain long-range nonlocal flow of a single row of vortices, which could be of interest for manipulation of individual vortices in quantum technologies,^37,38^ and allow tuning the value of the critical current by means of a gating voltage.^33^ On the other hand, three-dimensional W–C nanostructures can be grown by Ga^+^ FIBID as freestanding pick-up loops coupled to a SQUID^39^ and can be designed to support unconventional vortex patterns.^40^ Recently, K. Lahabi and co-workers have characterized the electronic and magnetic-field dependent properties of JJs created by FEBID.^22^ Superconducting properties of planar and 3D superconducting NWs based on Nb–C nanodeposits and fabricated by FEBID and Ga^+^ FIBID have also been studied,^41,42^ with 3D nanowires exhibiting a higher critical temperature than their 2D counterparts.

The vast knowledge on the properties of W–C and on how to tune them as desired, together with its commercial availability, make the W(CO)_6_ precursor the perfect candidate for a broad range of approaches for the fabrication of nanoSQUIDs. In a single-step process, a combination of normal- and superconducting materials can be used to fabricate SQUIDs based on both SNS- and SsS-JJs.

In this work, we present a method to nanofabricate W–C based dc-SQUIDs with two nanobridges by means of focused Ga^+^ ion beam induced deposition on flat Si/SiO_2_ substrates. Section 2 describes the instruments and parameters used to carry out the experiments. Section 3.1 outlines the fabrication process that we have developed to fabricate nanoSQUIDs in a single writing step. In Section 3.2 and 3.3 the results of the characterization of the electric and magnetic properties of the dc-SQUIDs are outlined.

## 2 Experimental

The devices were grown on Si substrates covered with a thermally-grown, 300 nm thick SiO_2_ surface layer. Prior to the deposition of the W–C nanoSQUIDs, a supporting Cr/Au structure, comprising the current and voltage leads for the electrical measurements of the devices, was patterned onto the substrates by optical lithography. A Süss MicroTec MA6 mask aligner, equipped with a 405 nm mercury lamp, has been used to transfer the design to a ∼2.8 μm thick MMA resist layer. An electron beam deposition system (BOC EDWARDS Auto 500) has been used to metallize the sample with a 5 nm Cr and a 50 nm Au layer followed by lift-off in acetone. The fine contacting structure was carried out *via* EBL in a Thermo Fisher Scientific Helios NanoLab 600 FIB/SEM microscope controlled by a Raith ELPHY Plus pattern processor to a 270 nm layer of PMMA resist. The metallization and lift-off steps were then repeated as described above.

The nanofabrication and imaging of the W–C SQUIDs were performed in the same Helios 600 NanoLab FIB/SEM microscope, fitted with a Ga^+^ ion column and a gas injection system (GIS) for precursor delivery. The imaging was performed with an electron beam current of 1.4 nA at an acceleration voltage of 5 kV. For the deposition of the W–C material, an ion beam current of 1.5 pA and an acceleration voltage of 30 kV were used. The volume per dose was 8.3 × 10^−2^ μm^3^ nC^−1^, the overlap was set to 50% and the dwell time was 500 μs. The base pressure of the FIB/SEM chamber was at 10^−6^ mbar, rising to 10^−5^ mbar during the injection of the W(CO)_6_ precursor gas. The nozzle of the GIS was positioned at a vertical distance of 50 μm and an in-plane displacement of 100 μm from the irradiation point.

The low-temperature characterization of the magnetotransport properties of the sample was performed in a commercial Quantum Design Physical Property Measurement System instrument. The base temperature for the characterization was 2 K. The samples were connected to the instrument *via* ultrasonic wire-bonding of Al wires between the Cr/Au leads and the instrument sample holder.

## 3 Results and discussion

### 3.1 Device fabrication

The fabrication of the nanodevices has been performed in a series of fully automatized sequential steps (Fig. 1), which include the fabrication of both the JJs and the main body of the nanoSQUID. In order to obtain the highest possible lateral resolution when depositing the nanowires, the lowest ion beam current of the FIB (1.5 pA) was chosen. The current has been kept at this value for the whole structure.

After the process chamber was flushed with the precursor gas for 20 s, two 50 nm-thick W–C large leads were deposited to carry the injected current from the Au leads to the device (Fig. 1a), taking 120 s. Thereafter, two 800 nm × 200 nm rectangular pads with a thickness of 50 nm were deposited in contact with the leads fabricated in the previous step, and positioned 300 nm apart from each other (Fig. 1b), taking 60 s.

During the deposition of materials by FIBID, a common problem is the undesired deposition of material in the vicinity of the irradiated area, an issue commonly referred to as halo. In the case of conductive deposits, the halo can carry part of the injected current. This is the reason why the halo deposit must be eliminated to ensure proper device functionality. We observed a significant amount of halo in between the pads, *i.e.* at the effective loop area of the SQUID. Thus the fabrication was paused until the precursor gas was completely evacuated from the chamber, taking 30 s, and a short FIB milling step of 2 s of the effective loop area was performed (Fig. 1c) in order to remove the unwanted metallic deposits inside the inner loop area of the SQUID. Upon gas injection for 20 s, two nanobridges were deposited connecting the two pads by their outer edges (Fig. 1d), taking 5 s. In total, the full fabrication process of the SQUID takes less than 3 min plus the time required to deposit the connections to the pre patterned gold structures, which strongly depends on their length. The nanowires have a cross-sectional area of 50 nm × 50 nm. The overall nominal loop area of the SQUID is 300 nm × 700 nm. The evaluation of the SEM images shows that the procedure is stable and highly reproducible with a low failure rate.

### 3.2 Superconducting properties

**Fig. 2:**
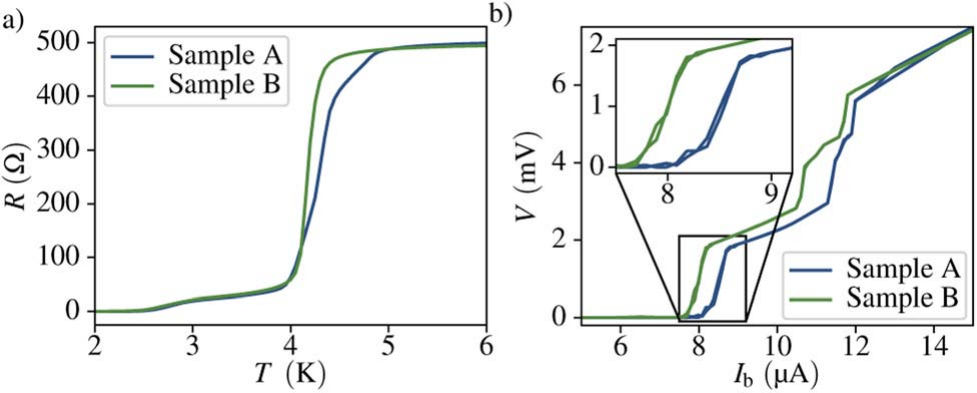
(a) Temperature dependence of the resistance in the region of the transition from the normal to the superconducting state. (b) *I*–*V* characteristics of the two samples. The different transitions are attributed to regions of different cross-sectional area.

In this section we present the superconducting properties of two identically grown SQUIDs, labelled A and B, in absence of an external magnetic field. The samples were cooled down to the base temperature of 2 K while constantly injecting a bias current *I*_b_ = 0.2 μA and measuring the resistance, *R*. The temperature dependence of the resistance is shown in Fig. 2a. Both SQUIDs exhibit a transition to the superconducting regime at *T*_c,A_ = 4.29 K and *T*_c,B_ = 4.17 K, respectively. This is in good agreement with the results found in literature.^34^

Thereafter, the current *vs.* voltage dependence was measured to obtain the critical current, shown in Fig. 2b. One can notice several transitions, attributed to both, the contact pads and the nanobridges. A quantitative analysis indicates that the critical current of the devices equals *I*_c,A_ = 8.513 ± 0.004 μA and *I*_c,B_ = 7.980 ± 0.004 μA for sample A and B, respectively. Although nanobridge SQUIDs are expected to exhibit hysteretic behavior due to dissipation of heat in the normal conducting state of the constrictions,^2^ we do not observe a hysteresis in the *I*–*V* characteristics. We attribute the suppression of the hysteresis and the high transition width to an increase of the effective temperature in the noise parameter *Γ* = *k*_B_*T*_eff_/*E*_J_ due to noise in the bias current.^1^ Here *E*_J_ = *Φ*_0_*I*_c_/2π denotes the Josephson energy. The normal state resistance of the full structure, *i.e.* above all transitions, is *R*_N,A_ = 496 Ω and *R*_N,B_ = 493 Ω for each sample. Both the critical current and the critical temperature are very similar for the two nanoSQUIDs, confirming the reproducibility of the fabrication procedure.

### 3.3 Critical current modulation

In a SQUID the quantization of the magnetic flux threading the loop requires the phase of the two JJs to fulfill the condition1
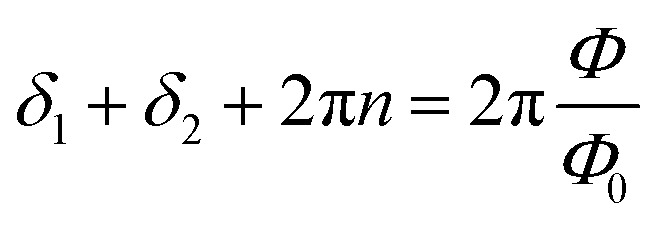
where *n* denotes the number of flux quanta and *δ*_i_ the phase shift in either of the nanobridges. The depth of the critical current modulation Δ*I*_c_/*I*_c,max_ in a SQUID is dependent on the dimensionless screening parameter2
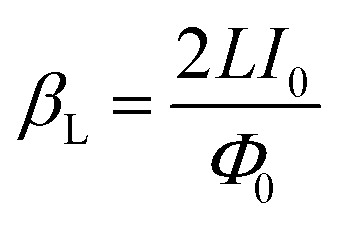
where *I*_0_ denotes the maximal Josephson current of a single junction. In the limit of *β*_L_ ≪ 1 the modulation depth becomes 1, in the limit of *β*_L_ ≫ 1 the modulation depth is reciprocally proportional to the screening factor, Δ*I*_c_/*I*_c,max_ ≈ 1/*β*_L_, allowing to estimate the SQUID inductance *L* from the modulation depth.^2^

**Fig. 3:**
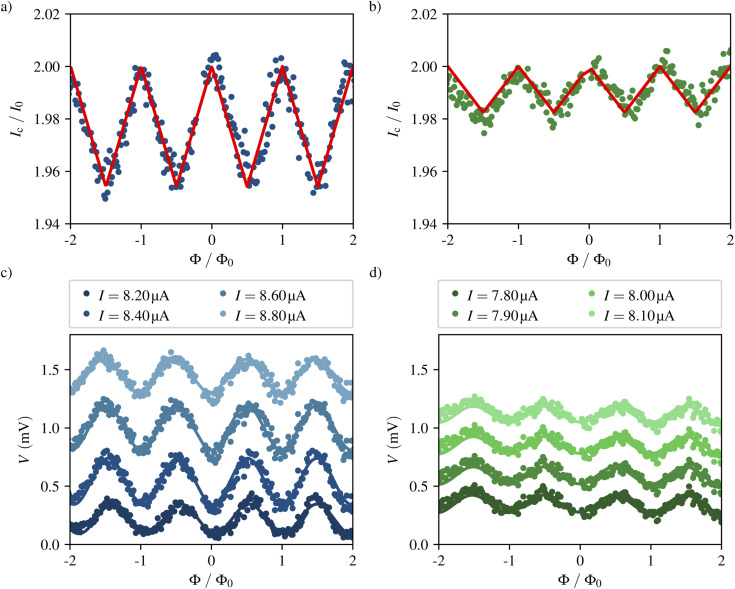
Electrical response of the devices to an external magnetic field at 2 K. (a and b) Critical current in dependence of the magnetic flux threading the SQUID loop for sample A (a) and B (b). *I*_c_ is normalized by the maximal Josephson current of a single junction. The red curve denotes the fit of the data to the model presented in eqn (3). (c and d) Voltage at various bias currents *I*_b_ ∼ *I*_c_ in dependence of the magnetic flux threading the SQUID loop for sample A (c) and B (d). In all plots the magnetic flux is normalized by the magnetic flux quantum *Φ*_0_.

To extract *I*_c_ we have measured *I*–*V* characteristics of the SQUIDs at different values of perpendicularly-applied magnetic field. As the magnetic flux threading the SQUID loop is quantized in integer multiples of the magnetic flux quantum *Φ*_0_, it is possible to attribute each period Δ*B* of the oscillation in *I*_c_(*B*) to a flux difference of Δ*Φ* ≡ *Φ*_0_. Fig. 3a and b respectively show the dependence of the critical current of samples A and B (normalized to *I*_0_) on the magnetic flux in units of *Φ*_0_. The low modulation depth of 2.3% in sample A and 0.9% in sample B, indicates a large screening factor. In this regime the critical current modulates as^43^3
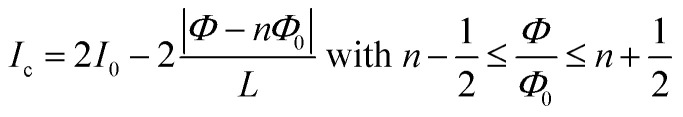


The red curves in Fig. 3a and b show the fit of this function to the data from which the following parameters are derived.

The period of the oscillation in *I*_c_ is similar in the two samples, with Δ*B*_A_ = 6.388 ± 0.016 mT and Δ*B*_B_ = 5.670 ± 0.026 mT. The effective areas *A*_eff_ = *Φ*_0_/Δ*B* deducted from this result are *A*_eff,A_ = 0.3238 ± 0.0008 μm^2^ and *A*_eff,B_ = 0.3647 ± 0.0017 μm^2^ and thus greater than the geometric loop area of *A*_geom_ ≈ 0.175 μm^2^. The effective area is in general higher than the hole size due to flux focusing from nearby electrodes as well as the high London penetration depth of W–C and the low thickness of the SQUIDs.

The SQUID inductance obtained from the fit is *L*_A_ = (10.4 ± 0.4) nH and *L*_B_ = (29.2 ± 1.4) nH, resulting in a screening factor (eqn (2)) of *β*_L,A_ = 43.5 ± 1.5 and *β*_L,B_ = 114 ± 6. As discussed in Section 3.2 the electronics used for the measurements inject a relatively noisy current, leading to a rounding of the *I*–*V* characteristics and suppression of the critical current.

### 3.4 Voltage modulation

The common operation mode of a dc-SQUID is its use as a flux-to-voltage transducer, where a constant bias current *I*_b_ ∼ *I*_c_ is injected and the voltage *V* is measured, exhibiting a sinusoidal dependence on *Φ*. In the vicinity of *Φ* = (1/4 ± *n*/2)*Φ*_0_ a linear dependence of *V* on *Φ* is obtained. The strongest variation of *V* for a change of *Φ* is characterized by the transfer coefficient4
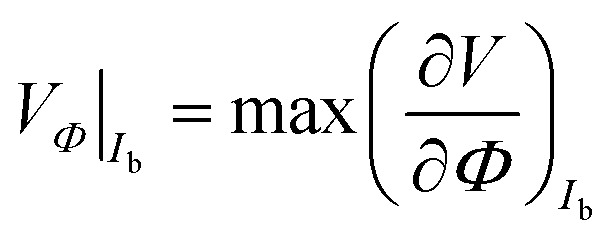


Thus, curves of constant *I*_b_ have been extracted from the *I*–*V* characteristics. Fig. 3c and d show *V*(*Φ*) curves for various values of *I*_b_ ∼ *I*_c_ of sample A and B, respectively. In sample A the transfer coefficient is *V*_*Φ*_ = 1300 ± 30 μV/*Φ*_0_ for a bias current of *I*_b_ = 8.5 μA. In sample B we obtain *V*_*Φ*_ = 473 ± 26 μV/*Φ*_0_ at *I*_b_ = 7.9 μA.

### 3.5 Comparison

The output voltage as a function of the magnetic signal of our SQUIDs is comparable to recent publications on Nb- and YBCO-SQUIDs. In 60 nm-thick Nb SQUIDs with an inner loop area of 400 nm × 400 nm a transfer function of 188 μV/*Φ*_0_ is measured at *T* = 4.2 K.^44^ In 50 nm-thick YBCO-SQUIDs with 65 nm × 100 nm a transfer function of 2200 μV/*Φ*_0_ is found at *T* = 4.2 K.^45^

## 4 Outlook

This fabrication procedure reported here serves as a proof of concept for the fabrication of W–C nanoSQUIDs by means of Ga^+^ FIBID. It has the prospect to be modified and augmented in various ways. The conduction regime (normal- or superconducting) of the W–C deposit can be controlled by deposition at various substrate temperatures (cryo-FIBID)^46^ or the use of an electron beam (FEBID) at different beam currents.^22^ Thus the nanobridges can be readily replaced by a non-superconducting, FIBID-grown metal, resulting in SNS junctions. Thereby the fabrication of planar instead of sandwich-type SNS-JJ based nanoSQUIDs could be realized. A metallic heat-sink or a shunt resistor can be also added to the SQUID in a similar manner.

In recent years the development of SQUID on tip (SOT) probes resulted in a new generation of Scanning SQUID Microscopes (SSMs) with unprecedented resolution and sensitivity for the mapping of the magnetic structure of a given surface.^15,47,48^ In this approach a SQUID is positioned on the tip of a pulled quartz tube *via* a three-step evaporation process. Lithographic methods require large, flat substrates and reach their limit with the high aspect ratio of the tip. The technique presented here poses a possible alternative approach for the fabrication of a SOT probe on commercially available Atomic Force Microscopy (AFM) cantilevers. The apex of the tip could be cut with the FIB and thereafter a SQUID could be deposited on the resulting flat area while maintaining the previously discussed flexibility in the SQUID design.

The comparably high inductance can be improved by a lower effective loop area and higher film thickness to enhance flux focusing and the fabrication of shorter nanobridges close to the coherence length. With Ga^+^ FIBID the feasible linewidth is at around 50 nm and the London penetration depth is 850 nm. Recent studies showed that both parameters could be improved by the use of He^+^ ions for the deposition of W–C nanowires. Nanowires with a linewidth down to 10 nm exhibit a London penetration depth of 400–812 nm^37,49,50^ making the material a promising candidate for the improvement of the process developed in this article. Further work towards the optimization of the noise and sensitivity of the W–C SQUIDs is underway.

## 5 Conclusion

In this work we have successfully fabricated two W–C nanoSQUIDs with an inner loop area of 300 nm × 700 nm in a fast Ga^+^ FIBID-FIB process (<3 min). The SQUIDs exhibit a critical temperature of around 4.2 K and a critical current of around 8 μA at 2 K. Albeit the London penetration length of W–C is higher than that of similar devices of other materials, we have clearly observed oscillations of both the critical current and the voltage in dependence of the applied external magnetic field. The transfer coefficient is comparably high with up to 1300 μV/*Φ*_0_, which we attribute to the high normal state resistance of the devices (∼500 Ω).

The versatility of FIBID facilitates a high degree of freedom in the geometrical dimensions of the nanostructures and the substrate supporting the nanoSQUID, possibly making the process an alternative approach for the fabrication of SOT devices.

## Author contributions

F. S. performed the sample growth and the magnetotransport experiments, analyzed the data and wrote the first draft of the manuscript. P. O. contributed to the sample growth, data interpretation and the writing of the manuscript. S. S. and J. M. D. T. got the funding, supervised the research and contributed to the data interpretation and the writing of the manuscript.

## Conflicts of interest

There are no conflicts to declare.

## Supplementary Material
